# Supplementary material for: Importance of ACE2 for SARS-CoV-2 Infection of Kidney Cells
Source: Biomolecules. 2023 Mar 3;13(3):472. doi: 10.3390/biom13030472 (PMC10046276; doi:10.3390/biom13030472)
Supplement: Supplementary file 1 [file biomolecules-13-00472-s001.zip › biomolecules-2175575-supplementary.pdf]

Supplementary Material to:

Importance of ACE2 for SARS-CoV-2 infection of kidney cells

Marie-Kristin Kroll, Sebastian Schloer, Peynaz Candan, Nadia Korthals, Christoph Wenzel, Hannah Ihle, Kevin Gilhaus, Kim Rouven Liedtke, Michael Schöfbänker, Beate Surmann, Rita Schröter, Ute Neugebauer, Gita Mall, Stefan Oswald, Stephan Ludwig, Ursula Rescher, Beate Vollenbröker and Giuliano Ciarimboli

Figure S1 shows mRNA expression levels of ACE2 and TMPRSS2 in AB8 podocytes after viral transduction. Quantitative PCR analysis was performed as described in Materials and Methods.

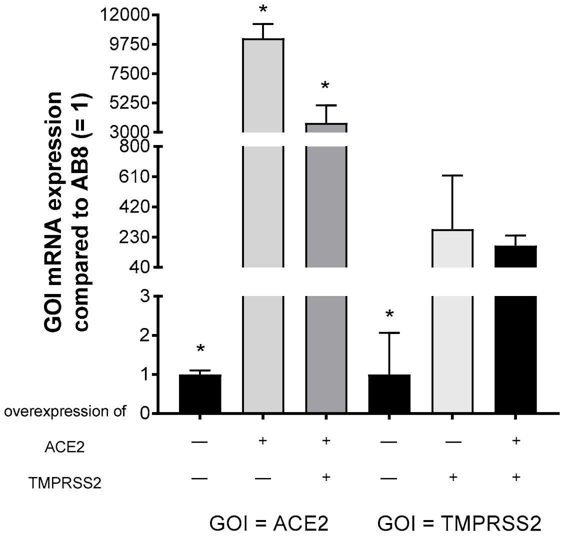

Supplementary Figure S1. Effect of viral transduction on expression of the genes of interest (GOI) ACE2 and TMPRSS2 in AB8 podocytes. This maneuver significantly increased mRNA expression of ACE2 both by overexpressing ACE2 alone or together with TMPRSS2. The ACE2 mRNA expression reached the highest level after transduction of ACE2 alone. TMPRSS2 mRNA expression significantly increased at the same level after transduction of TMPRSS2 alone or together with ACE2. N = 3 everywhere.

Figure S2 shows Western blot analysis of ACE2 and TMPRSS2 in human kidneys, Madin Darby Canine Kidney (MDCK) cells, and in AB8 podocytes after viral transduction.

Western blot analysis of lysates from human kidneys and AB8 human podocytes. For Western blot analysis, 2x Laemmli buffer containing sodium dodecyl sulphate (SDS) and ß-mercaptoethanol was added to cells grown to confluency. After this, cells were detached from plates by a cell scraper and the suspension was subsequently passed through a cannula at least 10 times. Thereafter, the solution was transferred to a 1.5 ml reaction tube and incubated at 95°C for 5 min. Lysates were shortly centrifuged before 10-15 µl of each sample was loaded on the stacking gel. Additionally, Precision Plus Protein Dual Color Standard (Bio-Rad, Hercules, CA, USA) was loaded on the gel as a molecular weight marker. Acrylamide gels (10%) of 1.5 mm thickness were used for protein separation. Electrophoresis was performed for 1 h at 150–200 V. The gel was then blotted for 1.5 h at 72 mA on a polyvinylidene difluoride (PVDF) membrane (Roche Applied Science, Mannheim, Germany). Upon completion of protein transfer, unspecific binding to the membranes was blocked by 1 h incubation in Tris-buffered saline with Tween 20 (TBS-T) and 10% (w/v) milk powder. Then, the membranes were cut (to control the loading by α-actinin staining) and incubated with primary antibodies at 4°C over-night. The following antibodies were diluted 1:1,000 in TBS-T with 5% BSA: α-actinin (Enzo Life Sciences, Farmingdale, USA); ACE2; Cell Signaling Technology, Danvers, USA); TMPRSS2 (Abcam, Cambridge, UK). After this, the PDVF membranes were washed and incubated for 1 h with goat-anti-rabbit or anti-mouse antibody (Dako, Hamburg, Germany) coupled with horseradish peroxidase (HRP) at a 1:2,000 dilution and washed again. Immuno-reactive bands were detected with an imager system (Azure c600, Azure biosystems, Dublin, CA, USA) by enhanced chemiluminescence using the reagent Clarity or Clarity Max Western blot ELC Substrate (Bio-Rad, Hercules, CA, USA).

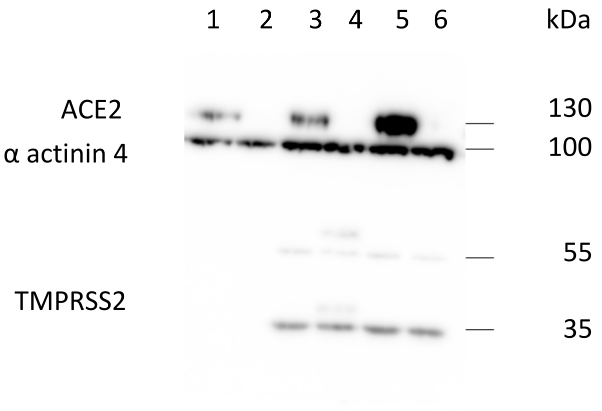

Supplementary Figure S2. Western blot analysis of ACE2 and TMPRSS2 expression in lysates from human kidneys (1), from Madin Darby Canine Kidney (MDCK) cells (2), from human immortalized podocytes AB8 overexpressing ACE2 (3), from AB8 overexpressing TMPRSS2 (4), from AB8 overexpressing ACE2 and TMPRSS2 (5), and from human immortalized podocytes AB8 (6). A band corresponding to ACE2 protein was detected at a molecular weight of ca. 130 kDa in human kidneys (lane 1) and in AB8 cells overexpressing ACE2 (lanes 3 and 5), but not in MDCK and AB8 cells. Probing the membrane with an antibody against TMPRSS2 produced no labeling in the human kidneys (1) and in MDCK cells, while two bands of around 35 and 55 kDa molecular weight were observed in AB8 cells. Interestingly, AB8 overexpressing only TMPRSS2 showed above these two signals at molecular weights of around 35 and 55 kDa an additional tiny band. Loading of samples on the membrane was visualized by antibody labeling of α actinin 4 (molecular weight ca. 100 kDa).

Figure S3 shows an immunofluorescence analysis of ACE2 and TMPRSS2 in AB8 podocytes after viral transduction. The analysis was performed as described in Materials and Methods.

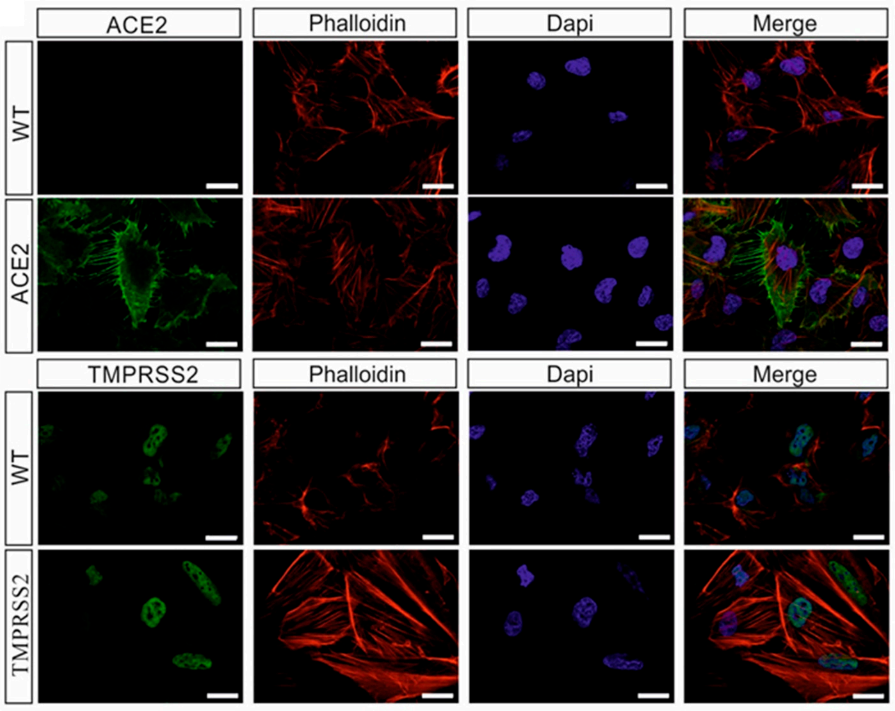

Supplementary Figure S3. Generation and characterization of stable cell lines. Immunofluorescence analysis of different AB8 cell lines showed an upregulated ACE2 expression (green color) in AB8+ACE2 cells which was localized within the plasma membrane. TMPRSS2 was found to localize within cell nuclei in AB8 WT and AB8+TMPRSS2 cells (green color), possibly due to unspecific antibody binding. Cell nuclei are visualized by DAPI (blue color) and the actin cytoskeleton by phalloidin (red). Scale bars indicate 20 μm.

Figure S4 shows the dependence of SARS-CoV-2 pseudovirus entry (using spike particles bearing Wuhan (W), Delta (Δ), and Omicron (O) spike variants) on ACE2 and TMPRSS2 expression in A549 cells wildtype (WT) or expressing either ACE2 (+ ACE2), TMPRSS2 (+TMPRSS2) or both + ACE2 + TMPRSS2). This analysis was performed as described in Materials and Methods.

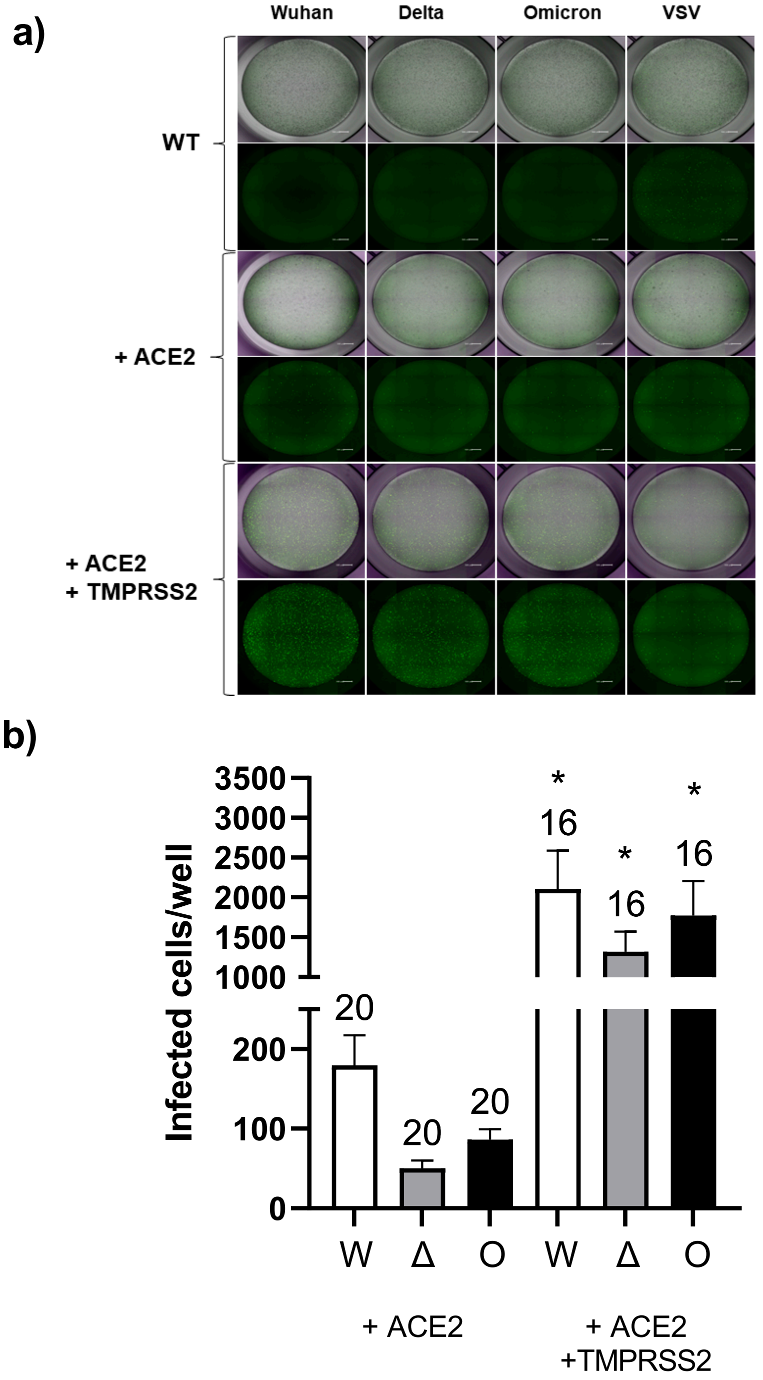

Supplementary Figure S4. Dependence of SARS-CoV-2 pseudovirus entry on ACE2 and TMPRSS2 expression in A549 cells. A549 cells expressing either ACE2 (+ ACE2), TMPRSS2 (+TMPRSS2) or both + ACE2 + TMPRSS2) were inoculated with VSVΔG/GFP-SARS-CoV-2 spike particles bearing Wuhan (W), Delta (Δ), and Omicron (O- variant BA.1) spike variants. GFP-positive cells were quantified.

Panel a) exemplary fluorescence images, panel b) quantitative analysis of GFP-positive cells. Cells were detected by staining the nuclei with Hoechst and were automatically counted. GFP-positive cells were automatically detected. The uptake of SARS-CoV-2 pseudoviruses was measured using a Celigo image cytometer (Nexcelom Bioscience, Lawrence, MA, USA). The vesicular stomatitis virus (VSV) was used as a positive control. For every cell line (WT, + ACE2, + ACE2 + TMPRSS2) the brightfield image (upper row) and the fluorescence image (lower row) are shown. Scale bars indicate 500 µm. No pseudovirus entry was detected in A549 WT cells. Overexpression of ACE2 resulted in a well-measurable number of GFP-positive cells, which was strongly increased by the concomitant presence of TMPRSS2. Data are given as mean ± SEM. The numbers above the columns indicate the number of independent experiments. * shows a statistically significant difference to all the other group ($p<0.01$, Anova with Tukey's multiple comparison test).
